# Complete genome sequences of six *Lactobacillus iners* strains isolated from Kenyan women

**DOI:** 10.1128/mra.00759-25

**Published:** 2025-10-16

**Authors:** Daniella Serrador, Landon J. Getz, Kevin Y. Cao, Jhenielle R. Campbell, Rupert Kaul, William W. Navarre

**Affiliations:** 1 Department of Molecular Genetics, Temerty Faculty of Medicine, University of Toronto 7938, Toronto, Canada; 2 Department of Biochemistry, Temerty Faculty of Medicine, University of Toronto 7938, Toronto, Canada; 3 Department of Immunology, Temerty Faculty of Medicine, University of Toronto 7938, Toronto, Canada; 4 Department of Medicine, Temerty Faculty of Medicine, University of Toronto 7938, Toronto, Canada; 5 Toronto General Hospital Research Institute, University Health Network 7989, Toronto, Canada; Wellesley College, Wellesley, Massachusetts, USA

**Keywords:** *Lactobacillus*, vaginal microbiome

## Abstract

*Lactobacillus iners* is one of the most common members of the vaginal microbiome, with a controversial role in vaginal health. Here, we present the complete genome sequences of six *L. iners* strains isolated from cervicovaginal secretions from women in Nairobi, Kenya.

## ANNOUNCEMENT

Most vaginal microbiomes are predominantly colonized by a single *Lactobacillus* species, such as *Lactobacillus iners,* which is estimated to be the most common vaginal bacterium globally (1, 2). Whether *L. iners* colonization protects against bacterial vaginosis is contested (1, 3, 4). Characterization of *L. iners* biology and the diversity of strains is needed to elucidate the role of *L. iners* in vaginal health. Here, we present the sequences of six *L. iners* strains isolated from Kenyan women.

Female sex workers in Nairobi, Kenya, were enrolled through the Sex Worker’s Outreach Program clinics. All strains presented here were isolated from *L. iners*-dominated vaginal microbiomes (determined by 16S rRNA gene sequencing), and each strain is from a different participant. Bacteria were isolated as previously described (5); in brief, frozen pellets of cervicovaginal secretions, collected using SoftCup (Evofem, San Diego, CA, USA), were plated on New York City III agar (6) and incubated anaerobically at 37°C for 48 h. All bacterial isolation and culture were performed in an anaerobic chamber (AS-580, Anaerobe Systems, Morgan Hill, CA, USA) using a gas mixture of 10% CO_2_, 10% H_2_, balance N_2_ (Linde, Mississauga, ON, Canada). *L. iners* isolates were identified using colony PCR for the inerolysin gene (5′-TACTAAGCCTGCACAAGCTG-3′; 5′-TGCATCAAATACATCACCTGG-3′).

For sequencing, 4 mL of Serrador’s *Lactobacillus*-adapted Iscove’s medium (5) was inoculated with an *L. iners* colony and incubated anaerobically at 37°C for 42 h. gDNA was extracted using the Wizard Genomic DNA Purification Kit (Promega, Madison, WI, USA, A1120), with the following modifications: 1.5 mL of culture was used, and for strains with low DNA yields, two preparations were combined during re-hydration. gDNA libraries were prepared using the Rapid Sequencing DNA V14 Barcoding kit (SQK-RBK114.24, Oxford Nanopore Technologies, Oxford, UK) using manufacturer’s instructions. Size selection was not performed. gDNA libraries were subjected to long-read DNA sequencing on a MinION Mk1B using Kit 14 chemistry (R10.4.1, Oxford Nanopore Technologies, Oxford, UK). All tools were run with default settings unless otherwise stated. Base-calling was performed using Dorado in duplex mode (v0.8.3, using the SUP model). Reads were demultiplexed and adapter trimmed using Dorado (v0.8.3, demux mode). Read quality and length were computed using NanoComp (v1.25.3) (7) (Table 1). Error correction was not performed, and no reads were removed during quality control.

**TABLE 1:** Genome sequencing, assembly, and annotation statistics per strain^*a*^

Strain	GenBank accession	Raw reads	Length (bp)	No. of reads	Avg read quality	Read length N_50_	Assembly coverage	GC content (%)	CDS	tRNA	rRNA	ANI
NVM020S14	CP194987.1	SRX29568733	1,354,189	52,819	13.8	4,861	53×	33.5	1,235	70	18	98.8
NVM025S01	CP173714.1	SRX29568738	1,359,327	86,416	15.2	3,901	79×	33.5	1,271	70	18	98.4
NVM041S27	CP194988.1	SRX29568734	1,319,338	351,366	21.8	1,298	203×	33.5	1,196	70	18	98.8
NVM076S08	CP194989.1	SRX29568735	1,305,175	419,916	20.8	1,132	260×	33.0	1,182	69	15	98.6
NVM196S05	CP194990.1	SRX29568736	1,340,659	12,535	12.9	6,476	70×	33.0	1,217	69	15	98.9
NVM210S01	CP194991.1	SRX29568737	1,353,401	607,684	20.8	932	285×	33.0	1,236	69	15	98.9

^*a*^ Read quality and read length were generated by NanoComp following sequencing (7). Assembly coverage was generated by Flye following assembly (8, 9). GC content, CDS, tRNA, and rRNA were determined after annotation using the NCBI Prokaryotic Genome Annotation Pipeline (10). ANI refers to average nucleotide identity as compared with the *Lactobacillus iners* reference genome (Strain: C0322A1, NCBI Accession: GCF_011058775.1), calculated by FastANI (11).

Genome assembly was performed with Flye (v2.9.5) (8, 9). Assembly coverage ranged from 53× to 285× across all six genomes, each a single circular contig (Table 1). Repeat graphs were manually visualized using Bandage (v0.9.0), confirming edge-to-edge coverage in genomes except NVM196S05 (12). NVM196S05 overlap trimming was done manually by mapping reads back to the assembly with minimap2 (v2.26-r1175) (13). Genomes were reoriented to begin at *dnaA*. The genomes were annotated using the NCBI Prokaryotic Genome Annotation Pipeline (v6.10) (10). Completeness and percent contamination were determined using CheckM (v1.2.3) (14). The average genome length was 1,338,681.5 bp with an average GC content of 33.25%. Each genome has an average nucleotide identity greater than 95% with *Lactobacillus iners* strain C0322A1, determined using FastANI (v1.33), confirming taxonomic assignment (11) (Table 1).

## Data Availability

The genomic data from this study have been deposited into NCBI’s BioProject under accession number PRJNA1184769. Accession numbers for both the assembly and raw reads are listed in Table 1.
